# Surgery

**Published:** 1893-07

**Authors:** 


### SURGERY.

EDITED BY DR. F. W. McRAE.

### AN IDEAL DRESSING.

The ideal dressing would seem to be a solution or paste which would quickly harden until it forms a thin flexible, impenetrable layer over the wound and surrounding skin, which would be thus hermetically sealed; in this way absolutely preventing any invasion of the wound from the outside, and preserving the aseptic conditions established at the operation. It would also be desirable, if possible, to add to these qualities the property of transparency.

allowing the line of the wound and the stitches to remain under constant observation, noting changes without disturbing the dressing. A dressing possessing such qualifications may certainly be named ideal.

My researches have been in a large measure rewarded; for although unable to secure a transparent dressing unaffected by cotton or other protective in contact with it, I have found, and for two years passed, used dressing which hermetically seals the wound in a thin layer, with certainty, preventing the invasion of pathogenic organisms from without. This dressing is easily made, simple, and always satisfactory.

After closure of the incision, the skin, the line of the wound, and the sutures are dried, and two layers of sterilized gauze or cheese-cloth, large enough to project five to ten centimeters (two to four inches) beyond the incision on all sides, laid on the skin. This is saturated with the following adhesive mixture, which is evenly distributed over the whole surface:

℞. Squibb's ether, or washed ether, and absolute alcohol, equal parts.
Bichloride of mercury, enough to make the solution 1-16000.
Anthony's snowy cotton, enough to make a syrupy consistence, added in small pieces, stirring.

As soon as this is poured over the wound, evaporation begins to take place at once, and the celluloidin hardens, gumming the gauze fast to the skin. To avoid delay in waiting for this to grow quite hard, and to prevent adhesion to the cotton applied above it, the whole surface is freely dusted over with a finely powdered mixture of iodoform and boric acid.

℞. Pulvis iodoformi, 4 grammes, or 1 drachm.
Acidi borici, 28 grammes, or 7 drachms.
M.—Exactissime. Sig.—Dust freely on wound.

This powder is of itself an invaluable protective. I use it constantly in obstetric cases, separating the labia and throwing it into the vagina, where it acts as a guard to the vaginal outlet against septic invasion from without.

The wound thus sealed with celluloidin gauze may be left untouched for a week or more, when the dressing should be softened with water, or more rapidly with ether, the gauze lifted off, and the stitches taken out.

If there are any signs of suppuration, as evinced by pain, local tenderness and redness, associated with elevated temperature, the dressing should be removed earlier, and the discharge of the stitch-hole abscess promoted in the usual way.

The purpose of cotton heaped up on the abdomen is now no longer protective and antiseptic; it merely serves the purpose of padding out the inequalities for the application of the bandage. Common cotton may be substituted for absorbent and prepared cotton by simply sterilizing it in the Arnold steam sterilizer.—*Prof. H. A. Kelley, Baltimore, in Exchange.*

---

### HOW TO ADMINISTER CHLOROFORM PROPERLY.

Mr. W. J. Cleaver gives the following valuable advice in regard to the administration of chloroform: Take your folded towel or cap of flannel stretched over a wire frame, and your chloroform bottle, graduated if you like, but this is of no consequence; let the temperature of the operating room be at least 65° F.; if your patient be one of the very nervous kind, give from half an ounce to an ounce of brandy an hour beforehand; put your catch forceps or your tongue forceps on the pillow beside you; pour a dose of chloroform, a drachm or two, on to the towel or cap and hold it, to commence with, two or three inches away from the mouth and nose of your patient, gradually bring it nearer, but never so near as to exclude air from mixing with the chloroform vapor; at the same time keeping your mind and eye on the respiration movements alone. If the patient shows any symptoms of struggling, let assistants take hold of his wrists and allow him to move his arms about as long as he does not interfere with the administration. If he endeavors to get up he must, of course, be restrained, but on no account let half-a-dozen dressers throw themselves upon him, their united weight probably coming near upon half a ton. What chance has free respiration in such a case? A little pressure on the shoulders will in ninety-nine cases out of a hundred be found to be quite sufficient; when a patient is forcibly held down with half a dozen big men on top of him, his struggle ends in a kind of nightmare, with perhaps a fatal shock at the close of it. Continue the administration quietly and gradually, without being put out of countenance by the repeated demands of the operator—to know whether he is not yet under; and such remarks as, I never knew a man take such a lot, put it close to his nose, what a time he is getting under, etc., etc. You are giving the chloroform, not the operator, therefore take no heed of him. When there is no conjunctival reflex, and a pinch on the skin of the abdomen bears no result, the pupil of the eye fairly contracted, and the breathing regular but perhaps stertorous, you can allow the operating surgeon to proceed.

Pay no attention to the operation, however interesting it may be; watch your patient's breathing only, and continue the chloroform when you see any signs of returning consciousness. If you do this, and this only, you will never have cause to regret it.—*Sheffield Medical Journal.*

---

### THE POINT OF ELECTION FOR THE INJECTION OF IODOFORM IN TUBERCULOUS HIP DISEASE.

Büngner, in the *Centralblatt für Chirurgie,* December 24, 1892, describes his method of using iodoform in tuberculous disease of the hip. He says: "It can no longer be contested that iodoform has a distinct anti-tubercular action, not only in cold abscesses but also in tuberculosis of the joints. Instead of following the method of Krause and making the puncture for the injection above the greater trochanter, he advises the method of Küster. The spot where the femoral artery passes over the brim of the pelvis is determined, and a line drawn from it to the apex of the greater trochanter; where this line crosses the sartorius muscle at its inner border is the point where the trochar is to be introduced. The joint at this point is not only comparatively superficial, but the capsule also is thin, and there is an overlying bursa, which, once in ten times, communicates with the joint cavity. A comparative test of the two methods of Krause and Küster on dead bodies gave for the former nineteen successes and six failures, for the latter twenty-five successes, the fluid being well distributed in the joint, and comparatively little around the outside. A hypodermic syringe of ten grams capacity, with a needle five to seven centimeters long, was used, five to ten grams of a 20 per cent. mixture with glycerine being injected and repeated in from one to four weeks.—*American Medical Magazine.*

---

### THE RATIONAL TREATMENT OF BUBO.

The author advocates early and complete extirpation of all buboes. He claims that when once suppuration has begun in the glands, and he believes that suppuration begins early, even as soon as the third day, the inflammation extends to the peri-glandular tisssue, and then the case becomes long and obstinate from the continued suppuration. It is his opinion that an early operation should be urged in all cases of bubo not distinctly syphilitic, and even in the latter where a tendency to inflammation exists. By thus operating we secure: 1st, possible security from constitutional sepsis; 2d, immunity from possible gland tuberculosis and secondary general infection; 3d, prevention of suppuration, chronic abscess and sinuses; 4th, avoidance of extensive tissue destruction and a resulting unsightly scar; 5th, an avoidance of serious interruption of the patient's vocation; 6th, a duration of healing measured by a few days or weeks, in lieu of the many weeks or months required by other plans of management; 7th, freedom from dangerous, infective or hemorrhagic complications.—*Times and Register,* March 11, 1893.

---

### THE TREATMENT OF PENETRATING WOUNDS OF THE ABDOMEN.

Lühre is reported (*Centralbl. für Chir.*, December 17, 1892) as concluding from 324 cases which he collected and tabulated, including those of McCormac, Coley and Morton, that in cases of undoubted perforation, laparotomy is indicated, but he does not recommend the diagnostic method of Senn, by the use of gas. The percentage of deaths in this series of cases, excluding vesical wounds, was 64. The mortality in 152 cases of shot wounds was 62.9 per cent., while for other forms of wounds it was 34 per cent. In those cases in which laparotomy was performed within the first twelve hours, the mortality was 58.2 per cent.; but in those cases in which it was delayed for a longer period, the mortality reached 79.5 per cent.; while the mortality in those cases in which the length of time was unknown was 52.4 per cent.—*American Journal of Medical Sciences.*

